# Impacts of Racket Handle Design on Table Tennis Topspin Forehand Rally Performance Among Beginner Players

**DOI:** 10.3390/sports13010022

**Published:** 2025-01-14

**Authors:** Ryushi Kumamoto, Ping Yeap Loh, Yuqi He, Enrico Ferlinghetti, Matteo Lancini, Tadashi Uno

**Affiliations:** 1 Graduate School of Design, Kyushu University, Fukuoka 815-8540, Japan; 2 Department of Life Design and Science, Faculty of Design, Kyushu University, Fukuoka 815-8540, Japan; 3 Faculty of Sport Science, Huaqiao University, Quanzhou 362000, China; 4 Department of Mechanical and Industrial Engineering, University of Brescia, 25128 Brescia, Italy; 5 Department of Human Movement Sciences, University Medical Center Groningen, University of Groningen, 9713 AV Groningen, The Netherlands; 6 Department of Medical and Surgical Specialties, Radiological Sciences, and Public Health (DSMC), University of Brescia, 25128 Brescia, Italy; 7 Center of Liberal Arts and Science, Sanyo-Onoda City University, Yamaguchi 756-0884, Japan

**Keywords:** active lifestyles, physical activity promotion, skill acquisition, sport pedagogy

## Abstract

Physical inactivity is a major global public health concern, and table tennis offers a low-impact, engaging way to promote physical activity across various age groups. However, many beginners struggle to maintain effective participation due to their lower skill levels. Therefore, the development and reinforcement of stable grip techniques is crucial because it will help beginners achieve sustainable improvements in performance. This will provide additional opportunities to increase physical activity, and therefore overall health, across all age groups. Thus, in this study, we investigate the effects of a prototype table tennis handle on the racket angle and performance of beginners. The prototype handle features a 20° tilt to assist the player in maintaining a stable topspin forehand grip during play. The participants were randomized into three groups, Groups A, B, and C, which used the prototype handle, standard handle, and practiced with the prototype but performed tests with the standard handle, respectively. The participants executed topspin forehand strokes in approximately 30 min of practice, and data on racket angles, swing mechanics, success rates, and ball landing positions were collected. The results showed that Group A exhibited a larger racket open angle and a smaller racket face Angle than the other groups. However, the groups showed no significant differences in hit positions or overall success rates. Our results suggest that although the prototype handle can influence racket angles and some performance aspects, individual differences and swing mechanics should be considered.

## 1. Introduction

Physical inactivity is a significant global public health concern, making the promotion of accessible and engaging physical activities essential. Table tennis is a versatile and increasingly popular sport across various age groups, offering health benefits such as improved cardiovascular fitness, hand-eye coordination, and agility [1,2]. Its low-impact nature ensures suitability for individuals of all ages and fitness levels [3]. However, effective participation in table tennis requires a certain level of skill is a barrier to most beginners to sustain long-term participation.

Despite the growing global popularity of table tennis, many beginners struggle to achieve consistent improvements. Therefore, mastering basic techniques such as forehand driving remains a significant challenge for amateurs. Common techniques, such as the topspin forehand, which refers to the movement of the forehand by swinging the racket forward and upward to complete the stroke [4], making the ball fly forward while generating forward rotation [5], is used as a warm-up rally before a practice or match [6]. The topspin forehand is a critical attack tool in table tennis that can increase the threat and variability of stroke play [7]. Therefore, mastery of forehand topspin is considered to be the hallmark that separates beginners from professionals [8] because beginners typically face difficulties in developing proficiency rallies in the early stages.

Skill development in table tennis stroke play depends on several factors, including mastery of correct techniques such as grip [9], posture [10], and swing mechanics [11], and consistent practice to reinforce these skills. Notably, the quantity and quality of practice, combined with the players’ knowledge, play critical roles in improvement. Equipment choice is equally crucial because selecting the right racket tailored to the player’s style enhances strength and compensates for weaknesses, thus further supporting skill advancement.

**Figure 1:**
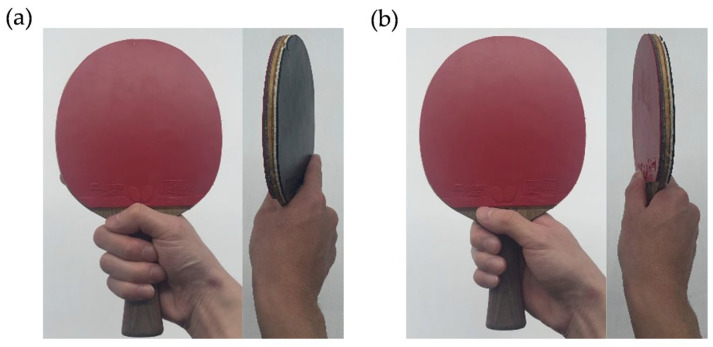
The typical grip: (**a**) forehand grip, (**b**) backhand grip.

However, among these factors, the grip technique directly affects key performance metrics, such as swing stability [10], racket control, and applied force [12]. Therefore, experienced players exhibit more consistent swing trajectories, stable racket angles, and precise grip pressures [13]. The widely used shakehand grip [14] is known as the forehand grip, which emphasizes firm holding with the middle, ring, and little fingers, and the backhand grip, which allows for greater wrist flexibility by loosening finger pressure (Figure 1). The forehand grip maintains a slightly open racket face, promoting consistent angles; however, the backhand grip enables versatile shotmaking through wrist movements.

Racket angle control is also pivotal in the shot trajectory and spin generation [15,16]. Instructional manuals highlight two critical aspects of racket control: the opening and closing of the racket face and its vertical orientation during strokes. Elite players maintain a forward tilt of approximately 35° during attacking strokes by adjusting the angle to generate speed or spin as required [17]. Therefore, increasing the forward tilt produces faster shots, whereas reducing it enhances spin, resulting in greater shot control.

Each grip style offers distinct advantages; however, beginners should develop a consistent forehand grip to maintain stable racket angles and improve shot consistency. Conversely, without proper instructions, novices often adopt ambiguous grip styles that lack the benefits of forehand or backhand gripping. Thus, even when taught the correct technique, many beginners still struggle to maintain their grip throughout play. Therefore, developing and reinforcing stable grip techniques are essential for overcoming these challenges and achieving lasting performance improvements.

The main objective of this study was to evaluate the differences in the racket angle between the prototype racquet and the conventional handle in forehand grip skill acquisition among inexperienced and casual table tennis players. We hypothesized that prototype handle would affect the racket angles during swings, and thereby influence the ball landing position and rally success rate.

## 2. Materials and Methods

### 2.1. Study Design and Participants

Twenty-four right-handed healthy university students were recruited for this study (Table 1). None of the participants received formal training or were regular players. Participants were randomized into three groups (A, B, and C), and each group was assigned specific handles for the practice and game sessions (Sessions I and II) to assess performance across different conditions. Notably, all participants were table tennis amateurs and had not played table tennis for at least a month before the experiment. The participants were informed of the experimental procedures, and they provided written informed consent before participation. This study was approved by the Research Ethics Committee of the Faculty of Design at Kyushu University (approval no. 541; 4 July 2023).

### 2.2. Experimental Environment and Handles Fabrication

**Figure 2:**
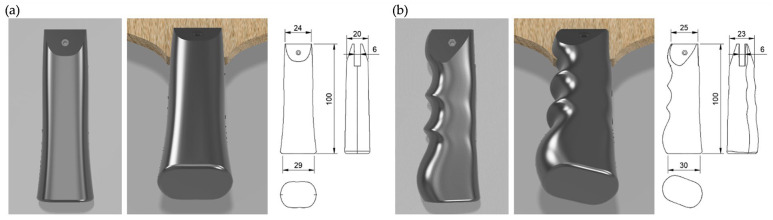
Dimensions (mm) of the (**a**) normal and (**b**) prototype handle.

The original handle of the beginner racket (GIANT, TSP, VICTAS Inc., Tokyo, Japan) was customized and adapted using the 3D printer’s original handle and the prototype handle (Figure 2) for practice sessions. The handles were composed of polylactic acid resin and fabricated using a 3D printer (Guider II 3D Printer; Flashforge, Tokyo, Japan). The surface was polished using waterproof sandpaper to prevent uneven contact, and all the participants used a standardized forehand grip posture (Figure 1a).

A wooden fabricated handle was used for each game session. Group A used the prototype grip exclusively during the practice and game sessions. However, Group B consistently used the original grips in both sessions. In contrast, Group C practiced with the prototype grip but switched to the original grip during the game sessions, enabling a comparison of the impact of grip transitions on performance. A standard table tennis table (B-2059, TOEI LIGHT Co. Ltd., Soka, Japan) was used in this study. Notably, the participants received no instructions from the researchers on how to swing or handle the rackets. This method allowed all participants to execute swing mechanics as novices without skill adjustments.

**Figure 3:**
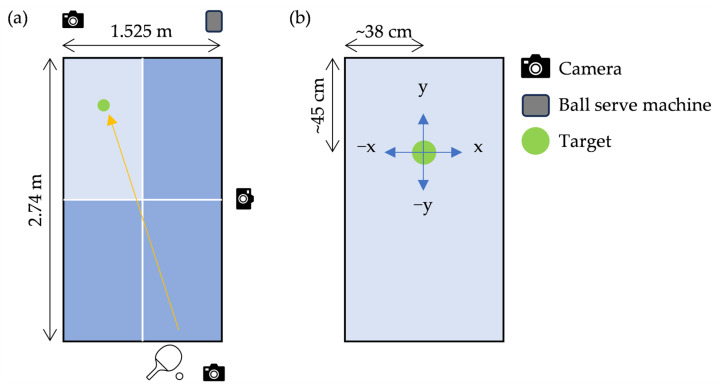
(**a**) Experimental layout, (**b**) definition of target and ball landed position with enlarged opposite court.

Notably, the participants engaged in a 30 min practice session before sessions I and II. Initially, the participants practiced the swing form and grip technique using beginner rackets (GIANT and TSP). Subsequently, they switched to the designated racket assigned to their group for machine drill practice. Participants continued practicing forehand rallies for 30 min, focusing on consistent returns to the designated target (Figure 3a). Therefore, to ensure stable foot positioning, a marker was placed at the tip of the toes of each participant to record their stance. If participants moved during the task, they were instructed to return to the marked position before the next shot.

In Sessions I and II, the main task involved achieving 10 successful returns from the ball machine. The participants were required to continue the task until they completed 10 rallies, and successful and failed returns were also recorded.

### 2.3. Video Data Acquisition, Data Processing, and Parameters Calculation

The experiment was conducted using a standard table tennis table (2.74 m long, 1.525 m wide, and 76 cm high) and a table tennis serve machine (Sakurai Boeki Co., Ltd., Amagasaki, Japan). Three high-speed cameras (Sports Coaching Cam ver.1.09, Logical Product Co., Ltd., Fukuoka, Japan) were placed perpendicular to the table at the front, back, and right sides. Figure 3 shows the experimental environment and setup.

**Figure 4:**
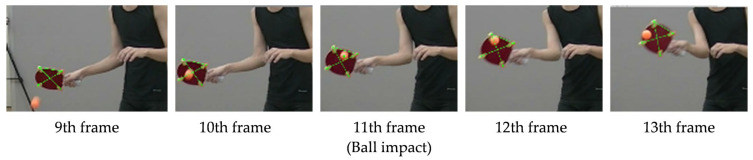
Image analysis during ball impact.

High-speed video cameras were synchronized to record movements and then converted to 60 fps using a high-definition Video Converter Factory Pro (Alice Fox Soft, Inc., Tokyo, Japan). Overall, 21 frames surrounding the ball contact event were extracted and analyzed. This included ten frames before and after the ball impact (11th frame), capturing the key moments before, during, and after the point of ball impact (Figure 4).

#### 2.3.1. Ball Landed Position and Success Rate

The position at which the returned ball landed on the table was calculated using a 3D direct linear transformation (DLT) method with Frame-DIAS V (Q’SFIX Co., Ltd., Tokyo, Japan). The exact moment at which the ball contacted the table was identified by digitizing the relevant frame. This allowed the calculation of the x- and y-planar coordinates, with the target point on the table defined as the origin (Figure 3). The success rate was determined by reviewing the video recordings captured from a front-facing angle: success rate = 10 (successful returns)/N (total number of attempts).

#### 2.3.2. Racket Open Angle (ROA) and Racket Face Angle (RFA)

The ROA and RFA were defined using the 3D DLT method with Frame-DIAS V. These angles were derived by digitizing the markers on the racket across 21 frames, capturing the moment of impact and 10 frames before and after the impact (Figure 4).

**Figure 5:**
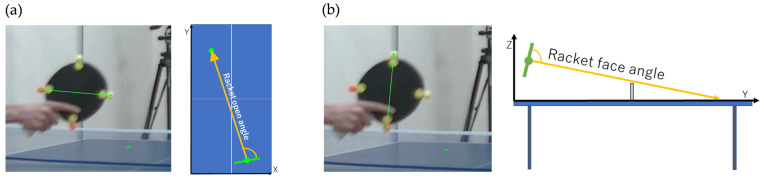
Definition of (**a**) racket open angle and (**b**) racket face angle.

ROA was defined as the projection of the line segment connecting the center of the racket to the target and the line segment connecting the markers on both ends of the racket to the x-y plane (Figure 5a). However, RFA was defined as the projection of the line segment connecting the upper and lower markers on the racket to the y-z plane (Figure 5b).

### 2.4. Statistical Analysis

Notably, all values are reported as mean ± standard deviation. A one-way analysis of variance (ANOVA) was performed to investigate the effects of handle design on the measurements. Pairwise comparisons using Tukey’s honest significant difference test were performed to determine the significant main effects. Furthermore, Spearman correlation analysis was used to reveal the relationship between racket opening and face angles, and racket opening and face angle changes over time (21 frames) with ball landing position and success rate. SPSS Statistics 24.0 (IBM Corp., Armonk, NY, USA) was used to perform statistical analyses, and statistical significance was set at α = 0.05.

## 3. Results

### 3.1. Ball Landed Position and Success Rate

**Figure 6:**
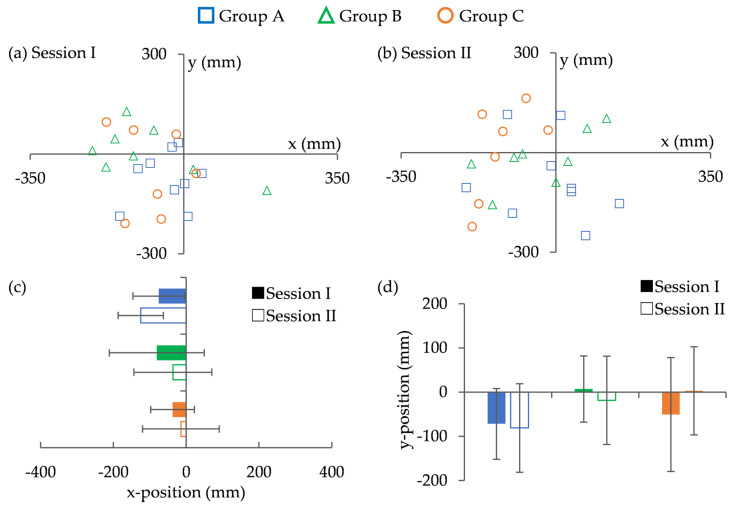
Ball landing position (**a**,**b**) composite landing location, (**c**) x position, (**d**) y position.

Figure 6 shows the average landing positions of the returned balls for each participant. The ANOVA results showed a marginally significant trend in the *x*-axis coordinates during Session II, with F(2,21) = 2.820, *p* = 0.082 (Figure 6). Post hoc comparisons indicated that Group A tended toward larger x-coordinate values than Group C, suggesting a greater horizontal deviation in the landing position. However, no significant main effects were found between the tasks in any group. These findings indicate that while group-level differences in the horizontal landing position were observed, there were no significant differences in performance across tasks.

**Figure 7:**
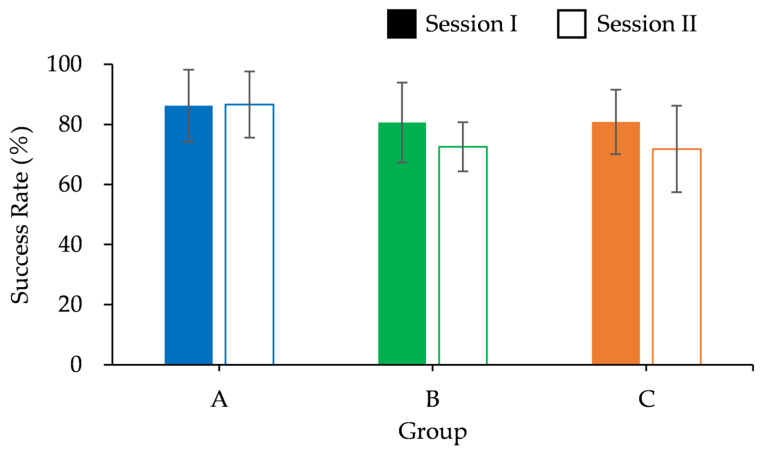
Success rate at Sessions I and II.

The average success rate for each group is shown in Figure 7. In both sessions, Group A demonstrated the highest success rate. However, the success rates of Groups B and C decreased during Session II.

Session II showed a marginally significant trend (F(2,21) = 2.788, *p* = 0.084) between the groups. However, post hoc comparisons did not reveal any statistically significant differences between the groups. Additionally, no significant main effects were found between the tasks in any group. These results indicate that, although Group A maintained higher success rates across tasks, the observed differences between groups and tasks did not reach statistical significance.

### 3.2. ROA and RFA

**Figure 8:**
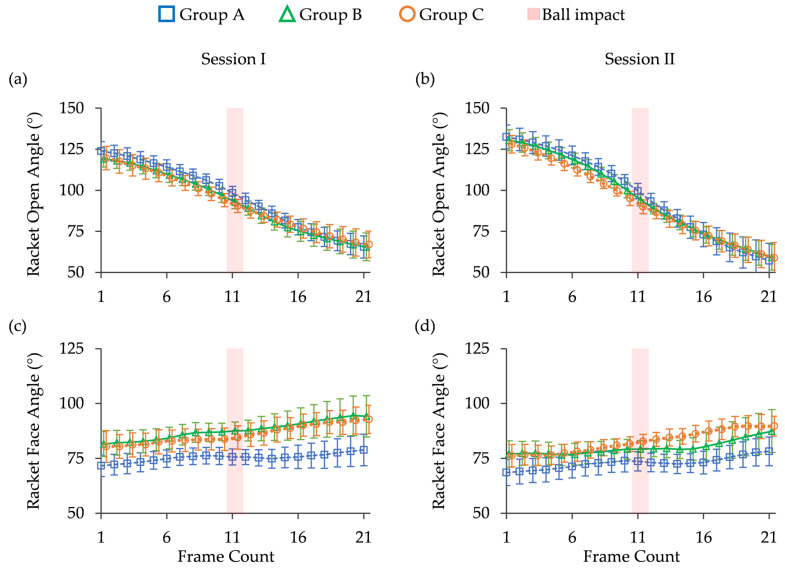
(**a**,**b**) Racket open-angle and (**c**,**d**) racket face angle at Sessions I and II throughout the 21 frames.

**Figure 9:**
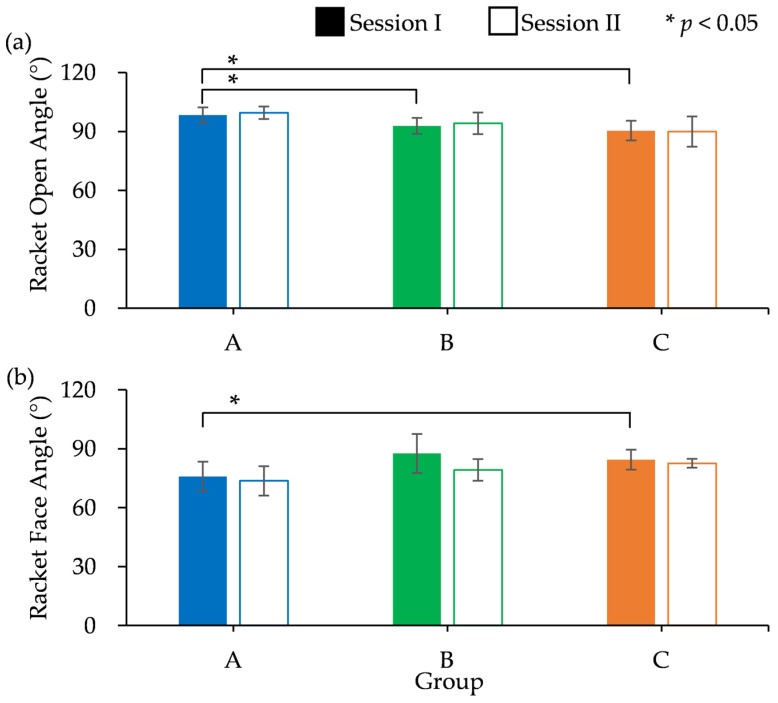
(**a**) Racket open-angle and (**b**) racket face angle at Sessions I and II.

Figure 8 shows the changes in ROA and RFA over time, and Figure 9 shows a comparison of the means between Sessions I and II for all groups. In Session I, the ROA had the highest value in Group A until the 16th frame, after which all groups had almost the same value in subsequent frames. The handle design had significant effects on the ROA (F(2,21) = 7.246, *p* = 0.004) and RFA (F(2,21) = 5.064, *p* = 0.016) during hitting. Furthermore, multiple comparisons of Tukey’s HDS results showed that group A had a significantly larger ROA than groups B and C and a smaller RFA than group B (Figure 9a).

In Session II, Group A showed the largest ROA until the 16th frame and had the smallest RFA. The handles had a significant main effect on ROA (F(2,21) = 5.940, *p* = 0.009) and RFA (F(2,21) = 6.094, *p* = 0.008) during hitting. Multiple comparisons showed that Group A had a significantly larger ROA and a smaller RFA than Group C (Figure 9b).

### 3.3. Relationship Between Racket Angles (ROA and RFA) on Ball Landing and Success Rate

A significant correlation between RFA on impact and success rate was observed only in Group C during Session I (r = –0.805, *p* = 0.029) (Table 2). Notably, no correlation was found in Group A, where the face angle was significantly smaller; therefore, it is considered that many participants in Group C swung with unique forms, differing from those in Groups A and B, as previously mentioned. Additionally, participants in Groups A and B consistently reproduced a face angle that kept the ball within the court, which explains the lack of correlation with the success rate.

Furthermore, no correlation was observed between ROA at impact and success rate under any condition. However, in Session I, a significant correlation between the success rate and changes in open-angle was found in Groups A (r = −0.834, *p* = 0.005) and B (r = −0.724, *p* = 0.042), and no significant correlations were observed in Group C. In contrast, in Session II, these correlations were present only in Group B (r = −0.713, *p* = 0.047) (Table 3).

## 4. Discussion

This study provides practical insights into handle design optimization for beginner table tennis players. We examined the effects of a prototype handle on the racket angles and the performance of beginners. Specifically, we analyzed the racket angles, namely the ROA and RFA, during impact, ball landing positions, and success rates of the rallies.

### 4.1. Effects of Prototype Handle on ROA and RFA

When examining the racket angles during Session I, a notable pattern emerged among the groups. Group A consistently displayed the largest ROA from frames 1 to 16 while simultaneously maintaining the smallest RFA compared with Groups B and C (Figure 8). This pattern was noticeable at the point of impact and during swinging. The notable variations between the groups imply that the racket angles of participants in Group A might have been affected by the prototype handle. Furthermore, the prototype handle might have encouraged Group A participants to adopt a handle and wrist position which facilitated better ball control during swings. This might have in turn influenced their shot consistency due to the larger ROA and smaller RFA.

In contrast, all groups showed a larger change in ROA across 21 frames than they did in Session I. This suggests that ROA increased more noticeably during the backswing and decreased more quickly during the follow-through. This phenomenon may have been caused by the participants taking larger swings after practicing forehand strokes. Notably, although Group C practiced using the prototype handle, there were no significant changes in the racket angle between the two sessions. Therefore, we postulate that the practice duration may have been insufficient for participants to adapt their swing mechanics to the prototype handle.

### 4.2. Relationship Between ROA and Landing Position

When examining where the ball landed, a notable trend emerged in Session II, such as in the shots of participants in Group A, which tended to land further to the right compared with those in Group C. This is likely to result from Group A’s significantly higher ROA. The participants did not show significant improvements in control after practice; however, there was a 55% reduction in the number of shots landing to the left of the target in Session II. This indicates a wider distribution of shots and suggests that the increased swing amplitude from practice affects the swing mechanics.

Notably, beginners tended to miss the left side of the target, regardless of whether their ROA at impact was > or <90°. Therefore, even with a larger ROA, over 70% of the participants missed the left. This implies an inherent tendency among beginners to veer left, possibly because of swing mechanics, lack of experience, or the body–table position [18].

However, in Session II, Group C showed positive correlation between the ROA and impact and landing positions. As the ROA increased, the shots landed closer to the target, indicating improved control. Group B exhibited a similar but less pronounced trend, while Group A showed no correlation. These differences suggest that practice during Session II might have reduced initial variability, leading to more consistent racket angles across participants. This phenomenon might have contributed to the lack of correlation between ROA and impact and landing positions in Group A, and likely to the low influence of ROA on landing positions.

Conversely, the absence of this correlation in Session I suggests that factors other than ROA, such as the swing direction and racket speed [8,13,19,20], affect where the ball lands. Research indicates that advanced players have consistent swing trajectories, whereas beginners exhibit significant variability [21]. Forehand topspin is a stroke technique that requires skill, making it difficult for developing players to control and adjust [8,22]. Therefore, even with an ideal ROA, swing inconsistencies can affect performance. Practice helps to reduce these individual differences, making the positive effects of ROA more apparent in Session II.

### 4.3. Relationship Between RFA and Landing Position

Notably, no direct correlation was found between the RFA at impact and landing positions under any condition. However, in Session II, significant correlations emerged between the changes in RFA during the swing and the composite landing position for Groups A and B. Specifically, greater changes in RFA were associated with shots landing closer to the target, suggesting better control.

This relationship may be influenced by swing mechanics. The changes in RFA were affected by the direction and speed of the swing. Studies have shown that advanced players execute swings with minimal variability [21] by moving the racket upward in a linear path and maximizing the swing speed at impact [7]. These ideal swing mechanics naturally increase the changes in the RFA, leading to improved control.

However, Group C did not exhibit this trend, implying that the changes in RFA were not due to ideal swing mechanics. These observations indicated that their swing patterns differed from those of the other groups, potentially affecting their ability to enhance control through changes in the racket angle.

### 4.4. Impacts on Success Rate

In the relationship between the ROA and success rate, a change in the ROA indicates the ability to maintain the angle of the racket toward the target. Therefore, we hypothesize that smaller ROA changes would be ideal for better control. However, these results contradicted our hypothesis. Consequently, we believe that changes in the open angle also reflect swing amplitude and speed, and swing speed was a greater contributing factor to the success rate than maintaining the open angle. Furthermore, the lack of correlation observed in Group C is thought to be due to the many participants swinging in unique forms, differing from Groups A and B.

### 4.5. Limitations

This study had several limitations. First, the practice duration might have been insufficient for participants to fully adapt to the prototype handle prior to data collection. Second, variability in individual swing mechanics among beginners, particularly in Group C, likely contributed to the inconsistencies in the results. Finally, the study focused solely on forehand rallies, whereas real gameplay involves a mix of forehand and backhand strokes. Future research should address these limitations by increasing the sample size, extending the training duration for adapting to the prototype, incorporating both forehand and backhand strokes, and examining a wider range of skill levels and playing conditions.

## 5. Conclusions

This study investigated the effects of a prototype racket handle on forehand grip performance among beginner table tennis players. The main findings indicated that participants in Group A who used the prototype handle maintained a larger racket open angle and a smaller racket face angle at ball impact across both sessions compared to other groups. These results provide a foundation for future research on racket handle design and its impact on skill acquisition.

## Figures and Tables

**Table 1:** Participant information.

	Group A		Group B		Group C	
	Male (*n* = 9)	Female (*n* = 0)	Male (*n* = 6)	Female (*n* = 2)	Male (*n* = 5)	Female (*n* = 2)
Age (years)	22.2 (21–23)	-	21.7 (21–23)	22.0 (21–23)	22.4 (21–23)	24.0 (22–26)
Height (cm)	172.4 (164.0–183.1)	-	171.2 (163.5–182.7)	154.4 (151.5–157.2)	169.6 (166.0–172.7)	153.9 (148.7–159.1)
Weight (kg)	59.2 (49.1–82.5)	-	59.3 (54.0–66.8)	43.5 (39.1–47.9)	51.3 (48.3–65.0)	43.3 (35.1–51.4)

Mean (min–max).

**Table 2:** Correlation between racket angles with landing position and success rate.

Session	Group	Racket Open Angle				Racket Face Angle		
		x Position	y Position	Success Rate		x Position	y Position	Success Rate
I	A	0.350 (0.356)	0.183 (0.637)	0.360 (0.341)		0.117 (0.765)	0.150 (0.700)	0.167 (0.668)
	B	0.333 (0.420)	−0.643 (0.086)	0.317 (0.444)		−0.619 (0.102)	0.452 (0.260)	0.073 (0.863)
	C	0.750 (0.052)	−0.107 (0.819)	0.430 (0.335)		−0.429 (0.337)	−0.571 (0.180)	−0.805 (0.029) *
II	A	−0.076 (0.847)	0.410 (0.273)	−0.092 (0.815)		0.326 (0.391)	−0.083 (0.831)	0.274 (0.476)
	B	0.690 (0.058)	0.762 (0.028) *	0.036 (0.932)		−0.204 (0.629)	0.084 (0.844)	0.037 (0.931)
	C	0.821 (0.023) *	0.857 (0.014) *	0.636 (0.124)		−0.143 (0.760)	−0.071 (0.879)	−0.455 (0.305)

r value (*p*-value), * *p* < 0.05.

**Table 3:** Correlation between racket angles changes over time with landing position and success rate.

Session	Group	Racket Open Angle Changes over Time				Racket Face Angle Changes over Time		
		x Position	y Position	Success Rate		x Position	y Position	Success Rate
I	A	−0.400 (0.286)	−0.317 (0.406)	−0.834 (0.005) **		−0.200 (0.606)	−0.183 (0.637)	−0.132 (0.735)
	B	−0.419 (0.301)	0.132 (0.756)	−0.724 (0.042) *		0.537 (0.170)	−0.708 (0.050) *	−0.250 (0.550)
	C	−0.214 (0.645)	−0.536 (0.215)	−0.430 (0.335)		0.643 (0.119)	−0.464 (0.294)	−0.412 (0.359)
II	A	0.151 (0.698)	0.100 (0.797)	0.513 (0.158)		0.147 (0.706)	−0.209 (0.589)	−0.275 (0.474)
	B	−0.527 (0.180)	0.048 (0.910)	−0.713 (0.047) *		0.429 (0.289)	0.524 (0.183)	0.218 (0.604)
	C	−0.143 (0.760)	−0.393 (0.383)	−0.655 (0.111)		−0.071 (0.879)	−0.643 (0.119)	−0.236 (0.610)

r value (*p*-value), ** *p* < 0.001, * *p* < 0.05.

## Data Availability

The data supporting the findings of this study are available from the corresponding author upon reasonable request.
